# Macrophage-Mediated Inflammation in Skin Wound Healing

**DOI:** 10.3390/cells11192953

**Published:** 2022-09-21

**Authors:** Alireza Hassanshahi, Mohammad Moradzad, Saman Ghalamkari, Moosa Fadaei, Allison J. Cowin, Mohammadhossein Hassanshahi

**Affiliations:** 1 Regenerative Medicine, Future Industries Institute, University of South Australia, Adelaide, SA 5095, Australia; 2 Department of Clinical Biochemistry, Faculty of Medicine, Kurdistan University of Medical Sciences, Sanandaj 66179-13446, Iran; 3 Department of Biology, Islamic Azad University, Arsanjan 61349-37333, Iran; 4 Vascular Research Centre, Adelaide Medical School, Faculty of Health and Medical Sciences, University of Adelaide, North Terrace, Adelaide, SA 5005, Australia

**Keywords:** macrophages, inflammation, wound healing

## Abstract

Macrophages are key immune cells that respond to infections, and modulate pathophysiological conditions such as wound healing. By possessing phagocytic activities and through the secretion of cytokines and growth factors, macrophages are pivotal orchestrators of inflammation, fibrosis, and wound repair. Macrophages orchestrate the process of wound healing through the transitioning from predominantly pro-inflammatory (M1-like phenotypes), which present early post-injury, to anti-inflammatory (M2-like phenotypes), which appear later to modulate skin repair and wound closure. In this review, different cellular and molecular aspects of macrophage-mediated skin wound healing are discussed, alongside important aspects such as macrophage subtypes, metabolism, plasticity, and epigenetics. We also highlight previous studies demonstrating interactions between macrophages and these factors for optimal wound healing. Understanding and harnessing the activity and capability of macrophages may help to advance new approaches for improving healing of the skin.

## 1. Introduction

Wound healing is a complex but finely-tuned process, which initiates immediately following injury and can continue for many months or years following wound closure. It is a multi-step process that requires coordination of four distinct but overlapping physiological stages which include hemostasis, inflammation, proliferation, and remodeling [1]. Wounds are divided into two categories of acute and chronic wounds. Acute wounds heal at a predictable and expected rate of healing, while wounds that fail to heal within 6 weeks and exhibit inefficient cellular and molecular functions are termed chronic wounds, which may lead to limb amputation if left without proper treatment [2,3,4]. It has been suggested that about 188 proteins are expressed more than twofold in chronic wounds, which may cause chronic inflammation, impaired angiogenesis, and dampened cell survival [5]. 

The inflammatory response is known as the first of several overlapping stages that constitute wound healing [6]. Inflammation has been reported to delay wound healing and cause increased scarring [6,7]. Macrophages, which constitute an important immunomodulatory cell type, play a key role in regulating inflammation and wound healing. They play important roles in protecting the host through multiple mechanisms such as phagocytosis, inflammation initiation and resolution, and growth factor secretion for cell proliferation and tissue recovery in wounds [8]. Macrophages release growth factors such as epidermal growth factor, keratinocyte growth factor, and tumor growth factor-α (TGF-α) to stimulate fibroblast and keratinocyte proliferation and production of collagen and extracellular matrix (ECM) proteins, leading to wound granulation and re-epithelialization [8]. In addition, macrophages secrete autocrine pro-resolving lipid mediators (SPMs) such as omega-6 (e.g., lipoxins) and omega-3 (e.g., resolving, protections, and maresins) [9]. SPMs regulate inflammatory responses and inflammation resolution. Interestingly, macrophages secrete IL-10, which prevents extra invasion of macrophages [10]. In addition, macrophages balance proangiogenic and antiangiogenic signals in wounds to manage angiogenic response during tissue granulation and scar resolution [11]. Generally, circulating monocytes migrate into the wound and differentiate into macrophages; these macrophages remove dead neutrophils through a mechanism named efferocytosis [12,13,14,15]. Along with monocyte-derived macrophages, resident macrophages concurrently stimulate inflammatory reactions by releasing hydrogen peroxide that attracts blood neutrophils and monocytes. Overall, migrating monocytes/macrophages and tissue-resident macrophages are believed to be the main regulatory cells that play critical roles in managing inflammation [16,17]. Therefore, a better understanding of how macrophages function in wounds can expand our current knowledge about macrophages’ contributions in the process of wound healing. 

## 2. Macrophages and Inflammation in Wounds

**Figure 1:**
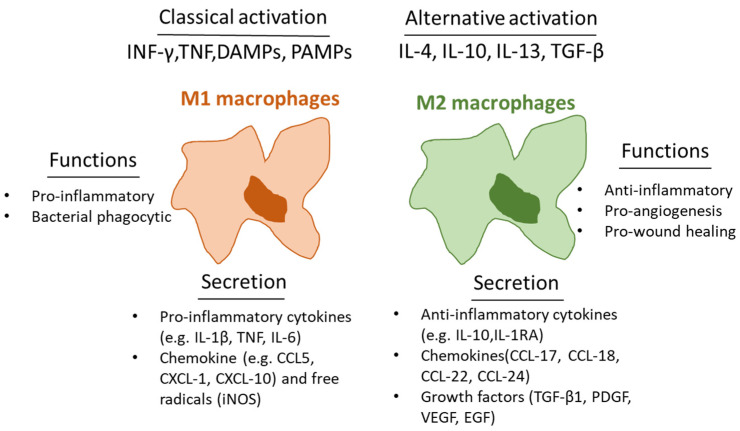
M1 and M2 polarization of macrophages. M1 macrophages produce pro-inflammatory cytokines, mediate resistance to pathogens, and possess strong microbicidal properties. M2 macrophages, on the other hand, are anti-inflammatory macrophages that mediate inflammation resolution and contribute to wound healing by promoting angiogenesis.

Skin macrophages arise from the two different developmental pathways: yolk sac-derived primitive hematopoiesis, which then develop to tissue-resident macrophages, and macrophages that originate from definitive hematopoiesis, which arises from aorta-gonad-mesonephros/fetal liver embryonically and bone marrow postnatally [18,19,20,21,22,23]. Macrophages possess distinct functional phenotypes as a reaction to microenvironmental stimuli and signals, referred to as macrophage polarization [24]. Macrophages polarity promotes or inhibits the inflammatory stage of wound repair [25,26]. In vitro wound healing studies have shown that macrophages are classically divided into two groups based on their phenotype and role: (i) the “classically-activated” macrophages, pro-inflammatory, or “M” (CD86^+^) macrophages that release cytokines including IL-12, IL-1β, IL-6, TNFα, and induced nitric oxide synthase (iNOS), and are involved in pathogen elimination, inflammatory cytokines release, and creating Th1-type reaction [27,28]; and (ii) the “alternatively-activated” macrophage, anti-inflammatory, or “M2” (CD206^+^) macrophages that promote angiogenesis, ECM repair, anti-inflammatory cytokines release, and inflammation resolution [29] (Figure 1). When macrophages phagocytose neutrophils, their phenotypes change from M1 to M2, a process regulated via mediators secreted from neutrophils [30]. In the inflammatory stage of wound healing, macrophages are attracted into the wound, where they present a polarity of M1 and M2 phenotypes that are regulated through cytokines, oxidants, lipids, and growth factors secreted by the macrophages [11,31,32]. Previous studies have suggested that macrophage plasticity plays a major role in wound healing [33]. As discussed later, macrophage plasticity is regulated epigenetically via histone modifications, DNA modifications, and microRNA; also, macrophage polarization is influenced through interaction with other cells such as adipocytes, infiltrating immune cells (polymorphonuclear neutrophils and T cells), and keratinocytes [33]. 

Previous studies have shown several medicators produced by macrophages which possess autocrine activity that can affect inflammatory response of M1 phenotype macrophages. Production of IL-1β and NLR family pyrin domain containing 3 (NLRP3) in macrophages are the main stimuli of inflammation and the M1 phenotype, leading to wound healing dysregulation [34]. Inflammation caused by macrophages requires two signals: a priming signal to transcript immature IL-1β [35] and a danger signal to produce mature IL-1β to release [34]. Glycoprotein Nmb (GPNMB) expressed by macrophages develop polarity of the macrophage from the M1 phenotype into the M2 [36]. It has been also reported that deficiency in Notch signaling induces high expression of TNFα, IL6, IL12, and iNOS, and increases inflammation through the effect on the Toll-like receptor and nuclear factor kappa B (NF-_k_B) pathways, as seen in diabetic conditions [37]. Another function of macrophage is the production of matrix metalloproteinases (MMPs), enzymes that degrade matrix and non-matrix proteins. MMPs are considered important modulators for switching the phenotype and function of macrophages [38]. For example, macrophages secrete a high concentration of MMP-9 (gelatinase-B) after invading the wound site [39]. This MMP can cleave macrophage integrin beta-2 (CD18) to switch the macrophages phenotype [40].

However, in vivo studies show that macrophages represent different features than just classic M1/M2 phenotypes seen in vitro [41,42]. While macrophages are classified using the broad F4/80 and CD11b markers, empirical evidence suggests the prevalence of multiple macrophage subtypes expressing combinations of macrophage specific markers with varied ontology. A seminal study by Tamoutounour et al. revealed the complex heterogeneity of skin macrophages [23]. This study identified a population of cells in healthy skin of dermal CD11b^+^ non-DC macrophages, including CCR2^−^ and CCR2^+^ cells. CCR2^−^ macrophages were further classified into Ly6C^Lo^MHCII^−^ and Ly6C^Lo^MHCII^+^ subsets, which were both CD64^Hi^MerTK^+^ and showed similar characteristics, including transcriptional profile, having foamy cytoplasm, and cell cycle kinetics similar to other tissue macrophages [23]. CCR2^+^ macrophages included Ly6C^Hi^MHCII^−^, Ly6C^Hi-to-Lo^MHCII^+^, and Ly6C^Lo^MHCII^+^ subpopulations, of which the latter two exhibited intermediate morphology between macrophages and dendritic cells. The differentiation of these subtypes was suggested to occur through CSF1R signaling. Transcriptomic and functional analyses demonstrated specialization of dermal macrophages. For instance, those with high phagocytic activity expressed the genes *C4b, CD209f, Tlr5, Pdgfc, Itga9*, and the scavenger receptors Stabilin-1 and CD36. Further analyses revealed dendritic cells developed from Flt3-dependent and CCR2-independent pathways. Ly6C^Hi^ blood monocytes generated dermal CCR2^+^Ly6C^Hi^MHCII^−^, CCR2^+^Ly6C^Hi-to-Lo^MHCII^+^, and CCR2^+^Ly6C^Lo^MHCII^+^ cells, whereas CCR2^−^Ly6C^Lo^MHCII^−^ and CCR2^−^Ly6C^Lo^MHCII^+^ subpopulations consistent of both embryonic and adult hematopoietic cells. Dermal wound macrophages actively and constantly alter their phenotype from pro-inflammatory to reconstructive [43,44]. For example, it has been recently shown that the level of CX3CR1 expression by macrophages play a critical role in wound healing [45]. This study categorizes macrophages into two CX3CR1^Hi^ vs. CX3CR1^Med/Lo^ subtypes, and suggest that a reduction of CX3CR1^Hi^ macrophages in type 2 diabetes leads to delayed wound healing [45]. Moreover, in in vivo wound healing, there is another category for macrophages, including tissue-resident macrophages vs. monocyte-derived macrophages [14]. Several studies classify M2 macrophages based on their function in the wound healing process, and sub-classify them into three macrophage subsets: M2a, M2b, and M2c. M2a is activated upon stimulation with IL-4 or IL-13, which subsequently results in macrophages secreting high concentrations of arginase-1, PDGF, insulin-like growth factor-1 (IGF-1), and other cytokines [46]. M2a also contribute to angiogenesis, proliferation, migration, and differentiation of fibroblasts [47]. M2b macrophages modulate anti-inflammatory and pro-inflammatory functions through the secretion of pro-inflammatory cytokines (e.g., TNFα, IL-6, and IL-1) and anti-inflammatory cytokines (e.g., IL-10 and IL-12) [48,49]. Therefore, this subset of macrophages can be a status between M1 and M2a polarity [50]. M2c macrophages have strong anti-inflammatory activity following stimulation with IL-10, TGF-β, or glucocorticoid [51,52,53]. Additionally, they contribute to angiogenesis by stimulating high endothelial cell migration and tube establishment [54,55]. They produce MMP-9 to absorb vessel and blood-derived stem cells in injured sites [56], phagocytize wound debris, and deposit ECM components [47]. 

An important point regarding the role of macrophages in wound healing is to know the contribution of tissue-resident macrophages and non-bone marrow-derived macrophages in modulating inflammation and wound healing. Currently there are insufficient studies to investigate the extent of the contribution of both bone marrow and non-bone marrow derived macrophages in wound healing. Previous studies have reported that chemotherapy and/or irradiation can cause significant bone marrow damage, leading to delay in hematopoiesis recovery and, thus, migration of monocytes into the circulation [57,58,59,60]. Therefore, it is important to interrogate whether non-bone marrow derived macrophages can compensate the delay in migration of circulating monocytes into the injury sites to regulate wound healing.

## 3. Macrophage Metabolism and Plasticity in Diabetic Wounds

The viability of immune cells is associated with their metabolism [61]. Therefore, it might be hypothesized that macrophage metabolism is altered in diabetic wounds. Macrophages utilize a different source of energy to produce adenosine triphosphate (ATP), and glucose has a pivotal role in orchestrating the ATP production in macrophages [62]. Glucose provides precursors for histone acetylation and methylation, which are known to be two major epigenetic processes altering macrophage plasticity and function [63,64]. Nicotinamide adenine dinucleotide phosphate (known as NADPH), which is important in producing reactive oxygen species (ROS), is generated within glycolysis [65]. Dysregulation of glucose metabolism is common in diabetes, resulting in changes in the number and type of macrophage-induced cytokines such as IL-1β, which leads to a dampening of glycolysis in macrophages in diabetic wounds [66]. Although the mechanism of compromised glycolytic capacity of macrophages is not fully understood, it seems that monocyte-driven macrophage function originating from bone marrow is highly affected by alterations in glucose metabolism in comparison with tissue resident macrophages [66]. In addition, the alternative activation of macrophages depends on others energy sources such as lipid synthesis and converting arginine to proline in the proliferation and remodeling stages of wound healing [62,67]. 

In diabetes, macrophages are known to have a pro-inflammatory phenotype, which is suggested to contribute to the pathogenesis of different diabetic complications [66]. This could influence macrophage metabolism in diabetes as M1 and M2 macrophage phenotypes rely on glycolysis, oxidative phosphorylation and tricarboxylic acid-dependent mitochondria in order to produce ATP [68,69]. It has also been hypothesized that ROS-induced mitochondrial damage in macrophages might prevent switching of M1 to M2 phenotype [70]. Furthermore, Zhang et al. demonstrated that in diabetes, improper functions of macrophages depend on glucose metabolism where under high glucose-availability, over activation of NLRP3 inflammasome is followed by increased expression of IL-1β, which subsequently leads to increased induction of M1 macrophages and elevated production of pro-inflammatory cytokines, which are detrimental for diabetic wound healing [71]. Membrane type 1 matrix metalloproteinase (MT1-MMP/MMP-14) promotes glycolysis in macrophages via hypoxia-inducible factor-1 (HIF-1) reported by Sakamoto et al. [72]. Here it was suggested that HIF-1 regulates oxygen availability in diabetic wounds and mediates macrophages metabolism [73,74]. Additionally, in diabetic wounds increasing IL-1β because of overexpression of TRL4 through mixed-lineage leukemia 1 (MLL1)-a histone methyltransferase at Histone H3K4 in promotor of TLR4 could change the metabolism of macrophage [75]. Although previous studies showed that glycolysis was used to produce ATP for M1 macrophages, glycolysis in general is a core physiological process that provides ATP for both M1/M2 macrophages [62]; thus, any changes in glycolysis and contributing factors are likely to change macrophage performance, and consequently affect healing of wounds in diabetes. Taken together, as glycolysis drives the metabolism of macrophages in diabetic and normal conditions, diabetes may cause significant alteration of macrophages’ metabolism, plausibly through alteration of glycolysis.

Compared to normal wounds, which transition from M1 (pro-inflammatory) macrophages to M2 (pro-healing) macrophages in a fine-tuned manner, diabetic wounds display dysregulated and persistent M1 macrophage polarization, resulting in prolonged inflammation and delayed wound healing [76]. Although the mechanisms by which macrophage function is altered in diabetes remain unclear, it is simplistic to consider hyperglycemia as the sole cause of disrupting macrophage plasticity in diabetes patients. In a study by Davis et al., it is suggested that the cyclooxygenase 2/prostaglandin E2 (COX-2/PGE2) pathway which regulates macrophage-mediated inflammation is highly activated in human and murine wound macrophages [77]. Using single-cell RNA sequencing of human wound tissue, this study showed (COX-2/PGE2)-mediated NFκB-activated inflammation of M1 macrophages. Another study showed that monocytes are exposed to oxidative stress, which consequently activate NF-κB via Toll-like receptor 2 in diabetic wounds [78]. Other studies suggested the important role of vitamin D in the downregulation of NFκB downstream signaling pathways, and showed that NF-κB-activated IL-1β, IL-6, and TNF-α pro-inflammatory cytokines were downregulated in diabetic mice [79]. Apart from NFκB, chemokine ligand2 (CCL2), a pro inflammatory chemokine, has been shown to be an important molecule that regulates macrophage function in diabetic wounds [80]. Studies have shown that the level of CCL2 in diabetic wounds negatively correlates with prevalence of M2-like macrophages. [81,82]. Similarly, others have reported that CCL2 is an important factor in maintaining the presence of M1-like macrophages in wounds [83,84].

## 4. Factors Affecting Macrophage Activity

**Figure 2:**
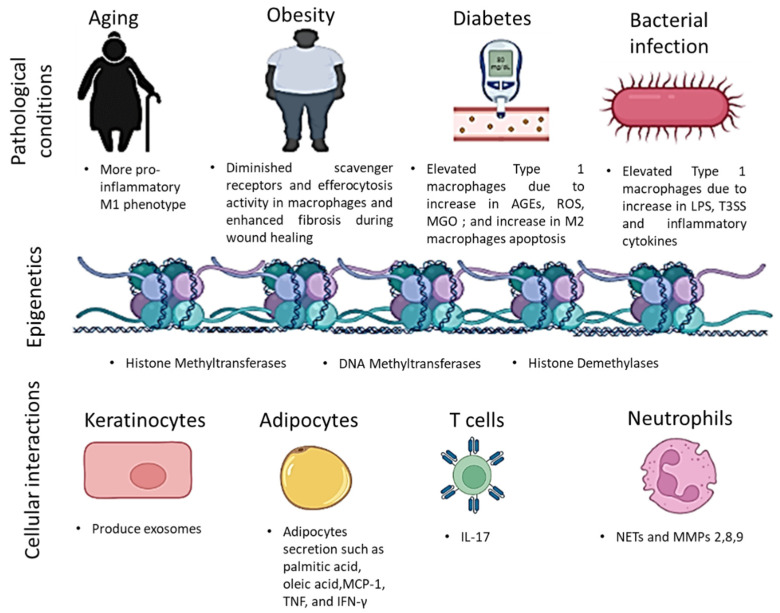
Different diseases and pathological conditions such as diabetes and obesity, epigenetic elements, and different cellular activities can induce inflammation by affecting macrophages functions which promote M1 macrophages activities.

During wound healing, the macrophage phenotype is regulated by epigenetic modifications (e.g., histone modification and DNA modification), miRNA activities, ATP-dependent remodeling, and cellular interactions, as addressed below and shown in Figure 2.

### 4.1. Epigenetic Modifications

Epigenetic regulators are involved in the processes of skin wound healing and are capable of dynamically regulating proliferation and migration of different cell types, including keratinocytes and endothelial cells [85]. As discussed here, studies have also illustrated that epigenetic factors regulate macrophages biology through a series of complex modulatory mechanisms that upregulate or downregulate gene activation to transiently alter cellular phenotype and function. 

#### 4.1.1. Histone Modification

Two additional histone modification events that play a significant role in the polarizing and switching of macrophages are methylation and demethylation. These occur via histone methyltransferases and histone demethylases. Histone methylation can activate or suppress transcription factors based on the position of the lysine and the number of the methyl groups added to the lysine residue [86,87]. MLL1 is one methyltransferase required for macrophage polarity, which increases the gene expression of proinflammatory macrophages during the inflammatory stage of wound healing. Further studies have shown that MLL1 knockout delays wound healing and reduces proinflammatory cytokines in a murine model of obesity and type 2 diabetes [88]. Two other main mechanisms that play roles in macrophage polarity during wound healing included histone acetylation and deacetylation. In the acetylation process, histone acetyltransferases transmits the acetyl groups from acetyl CoA to the lysine residue on the histone tail. This process influences the relationship between the DNA and histone, and leads to gene expression [89]. A histone acetyltransferase called males absent on the first (known as MOF) increases in type 2 diabetes condition, and promotes inflammatory genes [90]. Sirtuin 1 (known as SIRT1), a class of deacetylase enzymes, controls macrophage inflammatory reactions by deacetylation of the IFN-regulatory factor 8 (IRF-8) [91]. Furthermore, sirtuin 3 affects macrophage polarity during inflammation of the wound [92]. Therefore, histone modifications play a major role in switching macrophages phenotype during wound healing.

#### 4.1.2. DNA Methylation

DNA methylation is associated with macrophage plasticity [93]. DNA methylation of the peroxisome proliferator-activated receptor (PPAR)γ1 promoter leads to raised numbers of M1 macrophages and decreased M2 macrophages in diabetes [94]. Yu et al. suggested that PPARγ1 decreases chronic inflammation through increasing the number of M2 macrophages [95]. Therefore, manipulation of PPARγ1 may have a significant effect in diabetic wound healing by transitioning M1 macrophages to M2 macrophages. Further studies in the domain of epigenetic mechanisms have revealed that methylation of specific sites in histones affect macrophage polarization, allowing macrophage alterations depending on environment and tissue site [96]. The activation of Jumonji domain-containing protein 3 (JMJD3) as histone demethylase could act in favor of activation of both M1 and M2 phenotypes [97]. Likewise, methylation of CpG islands is involved in macrophage polarization in which the activation of both M1 and M2 phenotypes are developed [98].

DNA methylation mainly leads to the suppression of transcription factors that bind to DNA. This process occurs via DNA methyltransferases (DNMTs) that transmit a methyl group to the cytosine ring of DNA. DNMT1 controls macrophage phenotype towards the M1 [99,100]. When DNMT1 is inhibited by 5-aza-2′-deoxycytidine, macrophages are induced to take on a more M2 phenotypesand reduce inflammation [100]. Interestingly, the level of DNMT1 increases in T2D disease in mice, potentially contributing to the prevalence of M1 macrophages’ prolonged inflammatory state, and DNMT1 inhibition was found to promote wound healing in the mice [99]. It has also been shown that DNMT3b increases in macrophages of diet-induced obese mice. The DNMT3b also induces macrophage polarity towards the M1 phenotype, and DNMT3b inhibition also induces macrophage polarity towards an M2 phenotype [101]. Therefore, DNA methylation influences macrophage polarity and contributes to wound repair.

### 4.2. miRNA Regulation

Apart from methylation as major epigenetic affecting macrophage plasticity, recent studies have illustrated that miRNAs have the potential to impact on macrophage performance in diabetic wounds [102]. In this regard, miRNA-497 has a protective role against pro-inflammatory cytokines such as IL-1β, IL-6, and TNF-α inducing prolonged inflammation in diabetic wounds where M1 phenotype dominates [103]. Moreover, miRNA155 promotes wound repair by enhancing M2 phenotype, and miRNA21 has been shown to have a multifunctional role in wound healing, affecting the inflammatory and remodeling phases [104]. This may be particularly important as miRNA21 is affected by hyperglycemia, a metabolic condition affecting healing of diabetic wounds [105]. In the early phase of injury miRNA21 can induce polarization of M1 macrophages under hyperglycemic conditions [106] and drives the transition to M2 phenotype in later stages of healing [107]. Overall, this suggests that hyperglycemia has an important role in macrophages plasticity which is mediated through alterations in epigenetic or molecular signature (e.g., miRNAs and inflammatory signals) of macrophages.

miRNAs alter macrophage polarity by affecting gene expression [108]. MiR-146a expression is reduced in M1 macrophages, while it increases in M2 macrophages and MiR-146a can inhibit pro-inflammatory cytokines and exert protective effects on macrophages [109]. The expression of MiR-155 induces an M1 macrophage phenotype and inflammatory response [110]. In diabetic wound macrophages, MiR-21 overexpression is linked with the upregulation of pro-inflammatory genes including IL-1α, TNF-α, iNOS, IL-6 and IL-8, and induces the polarity of macrophages towards M1 phenotype [105]. 

### 4.3. ATP-Dependent Remodelling

Recent studies have shown that nanoliposome-encapsulated-ATP can improve wound healing [111,112,113]. This treatment affects macrophage polarization, progenitor cell recruitment, leukocyte chemotaxis, increased platelet, increased monocyte activity, monocyte differentiation to macrophages, increased macrophage proliferation, changes in RNA expression patterns, enhance collagen production by fibroblasts, and balancing between cell proliferation and regression. All cellular processes involved in wound healing require consuming cellular energy [114]. Thus, impairment in intracellular ATP can disrupt wound healing and lead to inflammation.

### 4.4. Cellular Interaction

The wound microenvironment can also regulate macrophage phenotype. Phenotype alterations of keratinocytes, adipocytes, T cells, and neutrophils have all been reported in diabetic wounds [115,116,117,118,119], which may affect their interactions with wound macrophages.

#### 4.4.1. Adipocytes

Dermal adipocytes can produce palmitic acid and oleic acid, as well as monocyte chemoattractant protein-1 and TNF. These adipocyte-produced biomolecules can change the macrophage inflammatory phenotype [90]. Palmitate can increase JMJD3 expression in macrophages that leads to the induction of inflammatory genes [90]. A study by Shook et al. showed that dermal adipocytes undergo lipolysis after injury, and contribute to skin wound healing through the recruitment of macrophages to the wound. This study further showed that adipocyte lipolysis impairment significantly compromised the number of macrophages in a wound, resulting in delayed revascularization and re-epithelialization of the wound bed [120]. Adipocyte-derived fatty acids and biomolecules can directly affect macrophages’ functions, as macrophages express multiple fatty acid receptors and transporters [121,122]. Intriguingly, previous in vitro studies illustrated that heat-inactivated, adipocyte-conditioned media can enhance monocyte/macrophage migration, suggesting that adipocyte-derived lipids may stimulate macrophage migration [123]. In addition, obesity-induced changes in macrophages and adipocytes lead to chronic inflammation and insulin resistance [124]. Obesity-related insulin resistance has been reported to correlate with elevated levels of pro-inflammatory cytokines such as TNF-α, IL-1β and IL-6 [125]. These cytokines are secreted by adipocytes due to increased release of pro-inflammatory factors during the development of obesity. These factors include free fatty acid, triglycerides, resistin, leptin, retinol binding protein 4, IL-6, TNF-α, and IL-1β [125]. These studies suggest that adipocytes can play a significant role in macrophage-mediated skin wound healing.

#### 4.4.2. Keratinocytes

Keratinocytes secrete different cytokines/chemokines and play an important role in cutaneous immunity. In chronic wound inflammation, keratinocytes release cytokines and interferons by regulating NF-_K_B, which affects immune cell inflammatory profiles [126]. In addition, increased proliferation of keratinocytes in chronic wound margins is observed compared to normal wounds [127]. A study by Villarreal-Ponce et al. showed that Ccl2 release by keratinocytes prompts macrophage trafficking and production of epidermal growth factor by macrophages in the wound [128]. Interestingly, macrophage-released epidermal growth factor stimulates keratinocytes proliferation. In a similar study, Zhou et al. showed exosome-mediated crosstalk between keratinocytes and macrophages in cutaneous wound healing. This study found that exosomes released by keratinocytes affected macrophage plasticity with pro-inflammatory macrophages exhibiting an M1 phenotype of pro-inflammatory resolution macrophages [129]. In vivo inhibition of keratinocyte-released exosomes resulted in a significant increase in the prevalence of pro-inflammatory macrophages in skin wounds [129]. This suggests that the fate and function of macrophages in the wound bed can be modified by other cell types, particularly keratinocytes.

#### 4.4.3. Immune Cells

Immune cells that infiltrate the wound are also shown to regulate macrophage phenotype in the wound healing process. Neutrophils are the first cells present in the wound that release neutrophil extracellular traps (NETs). In diabetic wounds, NETs are present at much higher levels than in normal healthy wounds. NETs induce inflammation and IL-1β secretion by macrophages [130]. In addition, growth factors and protease activity (i.e., matrix metalloproteases 2, 8, and 9) are elevated due to increased levels of neutrophils, serine elastase, and inflammatory macrophages, leading to prolonged inflammation [2,35,83,131]. Also, lymphocytes are known to play an important role in macrophage polarity [132]. It has been shown that T cells, especially gamma, delta, and Th17 cells, increase in numbers in diabetic wounds [133]. Th17 cells produce IL-17 that can regulate macrophage polarity. IL-17 elimination ameliorates wound healing in a diabetic mouse via decreased M1 macrophages and increased M2 macrophages [134]. These studies suggest that infiltrated immune cells in wounds can influence macrophage polarity.

## 5. Recent Studies and Conclusions

A better understanding of how macrophages function in wounds can provide better therapeutic approaches for skin wound healing. In a study by Theocharidis et al. it has been shown that murine macrophages or their secretome delivered in alginate dressings enhance impaired wound healing in diabetic mice [135]. In clinical practices, a study by Mao et al. discusses recent advances in biomaterials that balance the phenotypes of macrophages in wound healing [136]. Moreover, it has been reported that wounds treated with macrophages illustrated better cell recruitment and enhanced transition of healing process from inflammation to tissue repair [137]. This has led to the development of a novel hypothesis, which suggests that controlling macrophages modulation and recruitment in wounds may provide a better therapeutic outcomes compared to the approach that inhibits macrophages activities [137]. Recent studies have examined whether changes in macrophages polarization can improve skin wound healing. For instance, recent studies show that exosomes-laden self-healing injectable hydrogel increased diabetic wound healing by modulating macrophage polarization to improve skin angiogenesis [138]. Similarly, others have investigated whether mechanical stimulation plays a vital role in regulating macrophage polarization in the wound healing context [139]. 

Although important in different stages of skin wound healing, macrophages possess diverse biological features that help both the development and resolution of inflammation in wound repair. Macrophage subtypes have different physiological features with different molecular signatures. These molecular differences can induce or prevent various biological activities including inflammation, angiogenesis, and skin re-epithelization. In addition, macrophage metabolism and plasticity are affected in different conditions, such as obesity, aging, and diabetes, in which the wound microenvironment is distinctly altered. Moreover, the activity of other cell types including keratinocytes, adipocytes, and other immune cells can affect macrophage functions in wound healing. Collectively, macrophages are key players in skin wound healing, and further studies are required to elaborate how targeting macrophages can effectively improve skin wound healing in different pathophysiological conditions. 

## Data Availability

Data is available upon request to the corresponding authors.
